# In Vivo Imaging of Cardiac Attachment of TcI and TcII Variants of *Trypanosoma cruzi* in a Zebrafish Model

**DOI:** 10.3390/pathogens14010025

**Published:** 2025-01-01

**Authors:** Victoria E. Rodriguez-Castellanos, Cristhian David Perdomo-Gómez, Juan Carlos Santos-Barbosa, Manu Forero-Shelton, Verónica Akle, John M. González

**Affiliations:** 1 Biomedical Sciences Laboratory (CBMU), School of Medicine, Universidad de Los Andes, Bogotá D.C 111711, Colombia; ve.rodriguezc@uniandes.edu.co (V.E.R.-C.); js.barbosa@uniandes.edu.co (J.C.S.-B.); 2 Laboratory of Neurosciences and Circadian Rhythms, School of Medicine, Universidad de Los Andes, Bogotá D.C 111711, Colombia; cd.perdomo10@uniandes.edu.co (C.D.P.-G.); v.akle@uniandes.edu.co (V.A.); 3 Biophysics Group, Department of Physics, Universidad de Los Andes, Bogotá D.C 111711, Colombia; anforero@uniandes.edu.co

**Keywords:** Chagas disease, parasitic disease, intravital microscopy, zebrafish, tissue attachment

## Abstract

*Trypanosoma cruzi*, the etiological agent of Chagas disease, is a parasite known for its diverse genotypic variants, or Discrete Typing Units (DTUs), which have been associated with varying degrees of tissue involvement. However, aspects such as parasite attachment remain unclear. It has been suggested that the TcI genotype is associated with cardiac infection, the most common involved site in chronic human infection, while TcII is associated with digestive tract involvement. Traditional models for *T. cruzi* infection provide limited in vivo observation, making it challenging to observe the dynamics of parasite-host interactions. This study evaluates the cardiac attachment of trypomastigotes from TcI and TcII DTUs in zebrafish larvae. Labeled trypomastigotes were injected in the duct of Cuvier of zebrafish larvae and tracked by stereomicroscopy and light-sheet fluorescence microscopy (LSFM). Remarkably, it was possible to observe TcI parasites adhered to the atrium, atrioventricular valve, and circulatory system, while TcII trypomastigotes demonstrated adhesion to the atrium, atrioventricular valve, and yolk sac extension. When TcI and TcII were simultaneously injected, they both attached to the heart; however, more of the TcII trypomastigotes were observed attached to this organ. Although TcII DTU has previously been associated with digestive tissue infection, both parasite variants showed cardiac tissue attachment in this in vivo model.

## 1. Introduction

One of the most intriguing aspects of parasitic infectious diseases is the attachment and tropism of the parasites, which can occur at different levels, such as in tissues, cells, and even subcellular compartments, as in the case of an intracellular parasite [1]. The molecular mechanisms underlying pathogen preference for specific tissues remain poorly understood but are probably multifactorial, including many pathways and interaction sites between host and parasitic molecules [2]. Tissue attachment is contingent on the genetic variability of the parasite, host genetic factors, and immunocompetence; furthermore, tropism can change throughout the course of a particular infection [3]. In Chagas disease, caused by *Trypanosoma cruzi*, the hemoflagellate parasite mainly resides in the cardiac tissue during chronic infection, but it can also be found in the central nervous system of chronically infected immunocompromised individuals [4]. Tissue attachment in Chagas disease is not well understood, and genetic variability has been described as one of the factors that influence *T. cruzi* infection and tissue tropism [2,3]. 

The infective form of the *T. cruzi* parasite is the metacyclic trypomastigote, which is found in the feces of the triatomine vector. This form arises from the differentiation of epimastigotes, another extracellular and flagellated stage of the parasite within the vector. The vector defecates near the biting site, and the parasite enters the host via the damaged skin or intact mucous membranes. Subsequently, the parasite attaches to and infects cells, primarily macrophages and dendritic cells, and potentially various other mammalian nucleated cells. Within these cells, the parasite loses its external flagella and replicates asexually through binary fission. The resulting amastigotes eventually transform into trypomastigotes, which burst from the infected cells and spread through the bloodstream to infect other tissues. In addition to vector transmission, it can also be transmitted orally by consuming food contaminated with the vector’s feces; also, infection can be via blood transfusions, transplants, vertical transmission, and laboratory accidents [1]. For successful infection, *T. cruzi* must adhere to the host cell membrane to pursue cellular invasion [2]. 

*T. cruzi* genotypes are classified as Discrete Typing Units (DTUs), and currently, seven DTUs have been described (TcI to TcVI and TcBat). Among them, TcI and TcII are considered the two major parasite lineages, whereas the subsequent TcIII to TcVI variants are derived from the TcII lineage, constituting independent subgroups [5]. TcBat was initially described in Chiroptera with subsequent reports of infection in humans. TcI is mainly found in Central America and northern South America, and it has been associated with cardiac disease, causing cardiomyopathy in 90% of the chronic symptomatic cases, being the most common cause of infectious myocarditis. In contrast, TcII has a more limited geographic distribution in the central and southern regions of South America and has been associated with gastrointestinal diseases, causing megaesophagus and megacolon in less than 10% of chronic symptomatic cases [6].

To date, *T. cruzi* has been studied in multiple animal models, including mice, dogs, rabbits, and zebrafish [7]. Animal studies have different purposes, either to study cell attachment, motility [8], disease development, and immunity or response to treatment [9]. In mammals, the parasite can enter through the skin and mucous membranes, and in humans, there is an additional oral infection route where the parasite invades through the gastric mucosa. In both infection routes, the binding of *T. cruzi* to host cells relies on interactions between parasite ligands and cellular receptors [2]. Several of these ligands, mainly mucins and glycoproteins, are involved in binding to carbohydrate residues on mammalian cells, with gp85/trans-sialidase being one of the primary molecules involved [10]. Inside the mammalian hosts, the parasite travels through the vasculature to its preferred infection site [11]. Few studies have been able to study the behavior of *Trypanosomas* spp. inside living organisms [12]. A recent study in zebrafish showed that *T. carassii,* a natural host of zebrafish, moves by passive dragging with the blood flow in small capillaries or by active swimming against the current [13]. We have previously established zebrafish larvae as a model to study the motility of *T. cruzi* and found adhesion of the TcI trypomastigote to the heart, although *T. cruzi* is not a natural parasite of zebrafish [8].

The aim of this study is to assess the attachment of the *T. cruzi* parasite to various zebrafish tissues, with a TcII isolate, in relation to a TcI isolate. We focused on the tissue adhesion of different genotypes, using an established zebrafish model for *T. cruzi* motility from our lab. To achieve this, we used microinjection of fluorescently labeled parasites and monitored their dynamics inside zebrafish using stereomicroscopy and light sheet fluorescence microscopy (LSFM).

## 2. Materials and Methods

### 2.1. Type of Study

This experimental study was approved by the Institutional Animal Care and Use Committee (CICUAL) through the application form with code C.FUA_14-017 at the Universidad de Los Andes in Bogotá DC, Colombia.

### 2.2. Fish Breeding and Maintenance

Adult fish were placed in breeding tanks, and three females per two males were used to increase the number of eggs. The next day, eggs were collected using a strainer and then transferred to Petri dishes filled with egg water (a solution with a concentration of 0.6 g/L in deionized water with 0.01 mg/L of methylene blue with a pH between 7.2 and 7.9). Approximately 50 fertilized eggs were placed in each Petri dish, dechorionated using surgical forceps, and then placed in an incubator at 33 °C. Although the optimal temperature for the fish was 28 °C, the temperature was increased to 33 °C to maintain the parasites in an environment closer to the mammalian temperature. Before injection, the larvae were immobilized on 1% agarose (Amresco Inc., Solon, OH, USA). 

### 2.3. Parasite Culture and Labeling 

**Figure 1:**
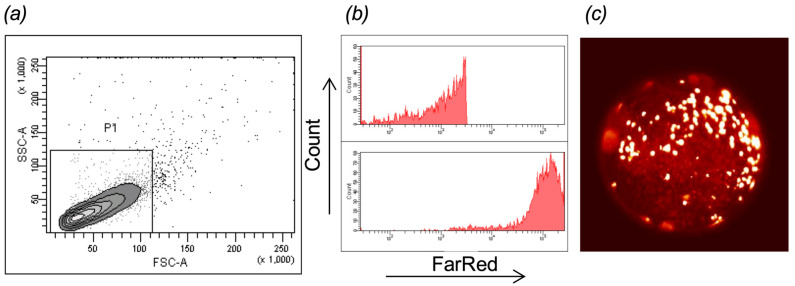
Detection of fluorescently labeled *T. cruzi* trypomastigotes. (**a**) Flow cytometry dot plot of forward scatter (FSC) versus side scatter (SSC) of TcI trypomastigotes. (**b**) Upper panel histogram of unlabeled TcI trypomastigotes as the control group, At 670 nm detection. Lower panel histogram of TcI trypomastigotes labeled with CTFR at 670 nm detections. (**c**) Standardized droplet of 160 μm diameter for microinjection under stereomicroscopy to estimate live parasites by their movement.

Isolated *T. cruzi* DA (MHOM/CO/01/DA) TcI and Y (MHOM/BR/00/Y) TcII were maintained in culture of a human astrocytoma cell line CRL-1718 (American Tissue Typing Culture, Manassas, VA, USA), in RPMI 1640 (Sigma, St. Louis, MI, USA) with 10% fetal bovine serum (FBS) (Eurobio, Les Ulis, France) incubated at 37 °C and 5% CO_2._ For labeling, trypomastigotes were collected from the culture supernatants, washed, and centrifuged for counting in a Neubauer chamber to obtain approximately 5 × 10^5^ to 1 × 10^6^ parasites per tube. The parasites were distributed in three different conditions: one for control (unlabeled) and the other for labeling with either CellTraceTM carboxyfluorescein succinimidyl ester (CTCFSE, Thermo Fisher Scientific, Cleveland, OH, USA) or CellTraceTM FarRed (CTFR, Thermo Fisher Scientific, Cleveland, OH, USA). The parasites were placed in 4 mL tubes, washed with 0.01 M PBS (1 × PBS) pH 7.4, and centrifuged at 1300 g for 5 min. The pellet was labeled with 1 mL of 1 × PBS containing 1 µL of CTCFSE or CTFR, incubated in the dark at room temperature for 10 min, washed with 1 × PBS, and centrifuged as described above. Parasite pellets were resuspended in 1 × PBS at a concentration of 100–200 parasites per 1 nL. For each condition, a sample was obtained for flow cytometry (FacsCanto II, BD Bioscience, San Jose, CA, USA) with FacsDiva software version 6.1. Fluorescence readings were performed at 510 nm for CTCFSE and 670 nm for CTFR PMTs. At least 10,000 parasites were examined under each condition. The gating and analysis strategies are illustrated in Figure 1. CTCFSE parasite labeling was also described previously [8].

### 2.4. Zebrafish Microinjection and Microscopic Follow-Up

Needles were pulled at 60 °C with borosilicate glass microcapillaries in a micropipette puller PC-10 (Narishige, Tokyo, Japan) with two light weights and one heavy weight. Each needle tip was observed and opened under a stereomicroscope and cut to 5–10 uM diameter with forceps. The needles were filled with 10 µL of the parasite solution at a concentration of about 100–200 trypomastigotes per nL and placed in a micromanipulator. For the microinjection, each larva was placed on an agarose mold containing egg water. The larvae were anesthetized with 0.002% tricaine solution (Fluka, Buchs, Switzerland) diluted in E3 water. Larval microinjection was performed in the duct of Cuvier, observed under stereomicroscopy, and the micromanipulator was placed at ~45° and parallel to the agarose mold. Once injected with 3–5 nL, each larva was placed separately in 6-well culture plates containing egg water. About 30 min after injection, zebrafish larvae were screened using a fluorescent stereomicroscope. An ET-Far Red fluorescence cube (filters: ET630/20x (EX), T647lpxr (BS), ET667/30m (EM); Chroma, VT, USA) was used to follow up CFTR-labeled trypomastigotes, while an ET-EGFP fluorescence cube (filters: ET470/40x (EX), T495lpxr (BS), ET525/50m (EM); Chroma, VT, USA) was used for CT CAFE-labeled trypomastigotes. Images and videos of each specimen were acquired using NIS-Elements software (Nikon, Tokyo, Japan). Then, larvae containing parasites adhered to any tissue of interest were transferred for follow-up using light-sheet fluorescence microscopy (LSFM). This occurred from 2 to 4 h after injection.

### 2.5. Detection by Light Sheet Fluorescence Microscopy (LSFM) 

We used a custom-made LSFM configuration as detailed [8]. Briefly, for the illumination arm, 488 nm and 633 nm diode lasers (06-MLD, Cobolt AB, Solna, Sweden) were used for CTCFSE and CTFR, respectively. A Galilean telescope was used to expand the laser beam, composed of f = 30 mm and f = 500 mm achromat lenses (Edmund Optics, Barrington, NJ, USA), and the expanded beam was then focused with a f = 75 cylindrical lens (Thorlabs, Newton, NJ, USA) into the back focal plane of an N Plan 10× NA0.25 air objective (Leica, Wetzlar, Germany), which was used to create the light sheet. For the detection arm, a water immersion CFI Plan Fluorite 40× NA0.8 objective (Nikon, Tokyo, Japan) and HQ525/50M and ET700/75 emission filters (Chroma, Bellows Falls, VT, USA) were used to capture the signals of CTCFSE and CTFR, respectively. Images were projected onto an sCMOS camera (Neo 4.5, Andor, Belfast, Ireland) using an f = 200 achromat tube lens (Nikon, Tokio, Japan). The injected larvae were mounted inside agarose-filled borosilicate glass capillaries and placed inside a chamber filled with egg water maintained at 33 °C. The intensity of light that reached each larva was set to 2 mW, videos were acquired at 50 ms exposure, and the sensor was cooled to −40 °C. The microscope was controlled with a custom LabView application (National Instruments, Austin, TX, USA), and ImageJ 1.53 software [14] was used to process and analyze the data. 

### 2.6. Injected Zebrafish Larvae Enzymatic Digestion for Flow Cytometry

The inoculated larvae were processed by enzymatic digestion for flow cytometry analysis. The procedure was conducted following Vargas-Patron 2019. In brief, anesthetized larvae were transferred to a 1.5 mL vial with calcium-free Ringer’s solution (NaCl 116 mM, KCl 2.9 mM, HEPES 5 mM) for 15 min. They were then transferred to a Petri dish, where 2 mL of 0.5% trypsin (Gibco, Waltham, MA, USA) solution was added. Then Petri dishes were incubated at 37 °C until larval cell dissociation was observed under a microscope. Trypsin was neutralized with 4 mL of a 5% FBS solution in 1 × PBS [15]. The solution was transferred to a 4 mL tube and centrifuged at 1300× *g* for 5 min. The pellet was resuspended in 250 µL of FACSFlow (BD Biosciences, Milpitas, CA, USA) and analyzed using a cytometer to detect the presence of labeled parasites. 

### 2.7. Data Analysis and Presentation

This was an experimental descriptive study. All videos and image stacks were captured using the aforementioned systems and stored in the raw format as they were acquired. The images selected for publication were converted to .tiff format to preserve quality, with no manipulation applied to any of the presented images. This study provides a qualitative description of the tissues to which the parasites attach, depending on their genotype. Consequently, inferential statistics were not used. The success of the injection was categorized as unsuccessful if no parasite was observed under stereomicroscopy, successful if any parasite was observed, and migration to an organ if a successful injection resulted in parasite migration and attachment to a specific organ. The four potential categories of tissue migration were classified as follows: eye or brain (due to proximity), heart or pericardium, GI tract, and circulation.

## 3. Results

Samples of labeled parasites in suspension were analyzed by flow cytometry to confirm their detection and later the specific fluorescence (Figure 1a). The flow cytometry histogram shows the absence of fluorescence in the control tube (Figure 1b, upper panel) and the fluorescence intensity at 670 nm when labeled with CTFR (Figure 1b, lower panel). A droplet of labeled parasite approximately 160 μm in diameter was standardized (Figure 1c) with injection pressures between 6 and 15 psi, depending on the needle used, to achieve this droplet diameter. Then, 178 larvae (48–72 hpf) were microinjected with either *T. cruzi* TcI (DA) (81 larvae), TcII (Y) isolates (64 larvae), or both simultaneously (33 larvae) into the duct of Cuvier, which is a part of the larval circulatory system. For the control, 1× PBS (the vehicle for the parasites) was microinjected. After 30 to 60 min, under fluorescence stereoscopy using a 40× lens (40× magnification), the migration and attachment of the parasite to different tissues and organs of the zebrafish were observed, from the duct of Cuvier onto the cardiac circulation, and from there to the cardinal vein (Video S1). The success rate of TcI injections was 91% (74 larvae), with organ migration observed in 28% (23 larvae) of the successful cases. For TcII, the success rate was 81% (52 larvae), with tissue migration occurring in 28% (18 larvae) of injections. Simultaneous injection of both TcI and TcII resulted in a 100% success rate (33 larvae), with the detection of either TcI, TcII, or both genotypes, and migration was observed in 30% (10 larvae) of the injections (Table 1).

**Figure 2:**
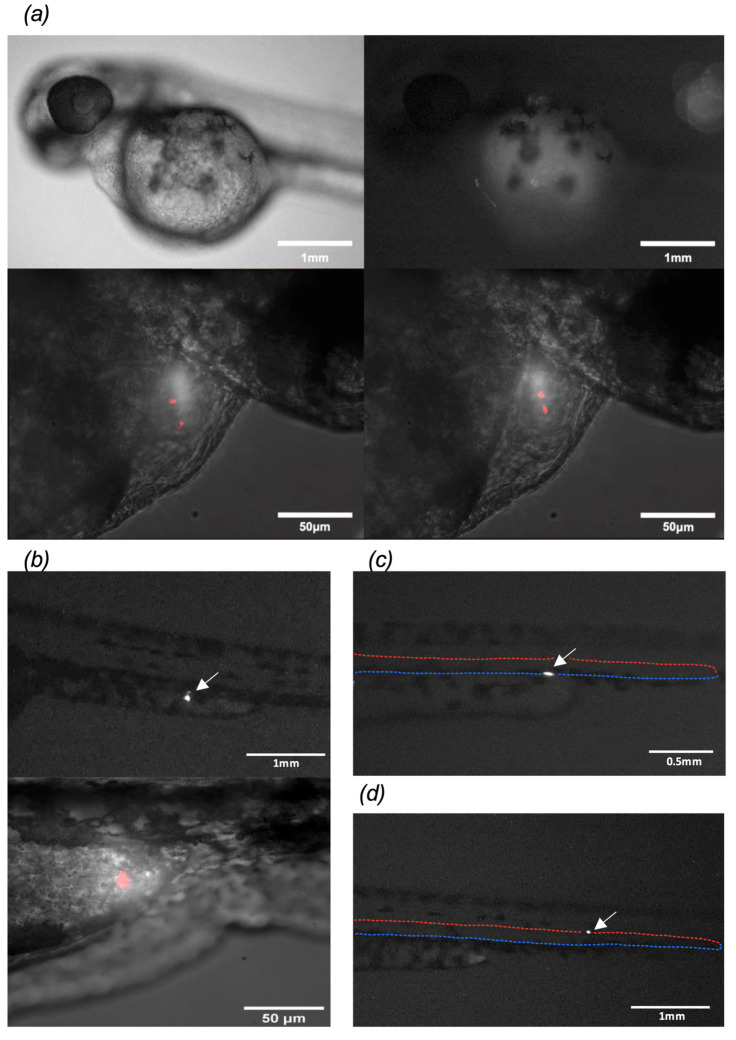
(**a**) Zebrafish larvae were injected with *T. cruzi* TcII trypomastigotes found attached to the heart valves of the larva under stereomicroscopy in the upper panel. Two trypomastigotes can be observed under LSFM 4 h after microinjection in the lower panel. (**b**) The upper panel shows the migration of the parasite towards the yolk sac extension under stereomicroscopy. The lower panel shows the same larva 4 h after microinjection under LSFM with a subtle movement towards the end of the yolk sac extension. (**c**) Zebrafish larvae injected with TcI trypomastigotes, one of which was attached to the cardinal vein (dotted blue line), observed under stereomicroscopy. (**d**) TcI trypomastigote attached to the dorsal aorta (dotted red line), observed under stereomicroscopy. The images shown came from different larvae.

Under stereomicroscopy at 40× magnification, parasites of the TcI genotype attached to the heart were observed to move with the heartbeat (Video S2). Using LSFM and at higher magnification, the parasites in the heart were found to be attached mainly to the heart valves and remained adhered to the atrioventricular valve of zebrafish with each heartbeat. Out of all the larvae injected with TcI, 35% were found attached to cardiac tissue (Table 2). In addition, TcII parasites were also found adhered to the cardiac tissue (Figure 2a). They were mainly found in the atrium, as observed under stereomicroscopy, which was later confirmed with LSFM (Video S3). Among all larvae in which TcII parasites were observed, cardiac parasite attachment was observed in 39% (Table 2). In the case of TcI trypomastigotes, up to two parasites were found attached simultaneously in different cardiac locations, whereas for TcII, it was possible to detect up to four attached parasites.

Using stereomicroscopy, TcII parasites migrating from the duct of Cuvier to the yolk sac extension, a structure that originates in the digestive tract of zebrafish, were observed (Figure 2b upper panel). These larvae were subsequently followed by LSFM, and detectable parasite migration towards the yolk sac extension was found (Figure 2a, lower panel). Remarkably, TcI adhesion to the yolk sac extension was detected in 22% of the larvae that were positive for parasite migration, while TcII adhesion was observed in 28% (Table 2). In two larvae with TcI parasites adhered to their hearts, trypomastigotes were also observed in the peripheral vasculature. This location was determined by transmitted-light stereomicroscopy, as shown in Figure 2b, where a parasite was observed attached to the posterior cardinal vein. Additionally, in another larva, a trypomastigote was detected in the endothelium of the dorsal aorta (Figure 2c). Attachment to the zebrafish peripheral vasculature was observed in 26% and 17% of cases for TcI and TcII, respectively (Table 2). 

**Figure 3:**
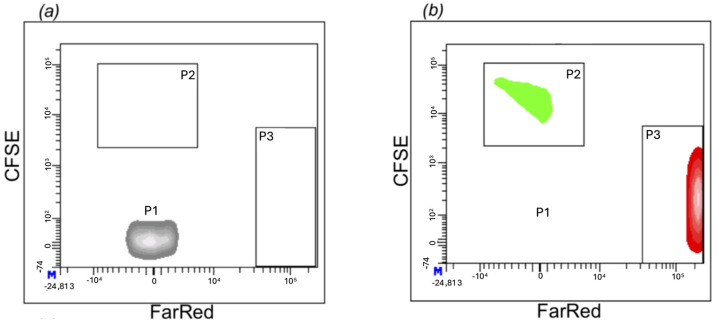
Dot plot of flow Cytometry assay with *T. cruzi* trypomastigotes. (**a**) The unlabeled population (P1) served as the control. (**b**) Region P2 represents TcII trypomastigotes labeled with CTCFSE, while region P3 represents TcI trypomastigotes labeled with CTFR.

**Figure 4:**
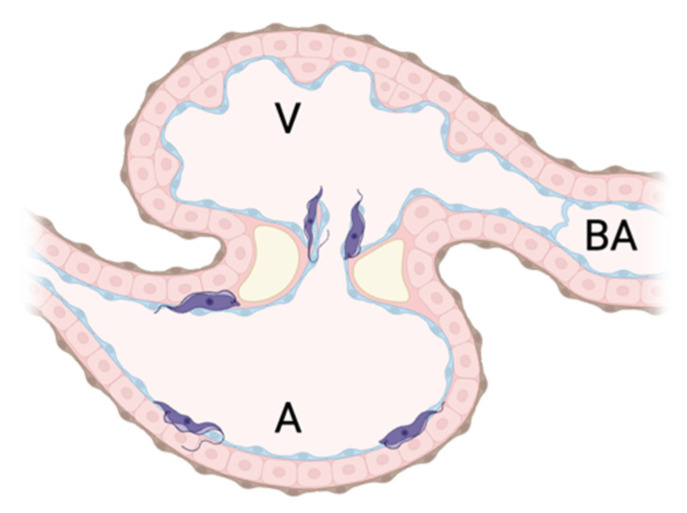
Schematic representation of zebrafish larvae heart showing the locations where trypomastigotes were detected using LSFM. Parasites (purple figures) were observed to be attached mainly to the heart valves and atrium, independent of DTU. V: Ventricle, A: Atrium, BA: Bulbus arteriosus. Figure was made using BioRender.com.

Parasite attachment of both DTUs was assessed by microinjecting them simultaneously into the same larvae (33 larvae) with different dyes in order to distinguish them. Trypomastigote labeling with each dye was confirmed by flow cytometry, and three populations can be distinguished: the population in region P1 without labeling, used as a control (Figure 3a); the population in region P2 consists of TcII parasites labeled with CTCFSE (CFSE-green channel); and the population in region P3, TcI labeled with CTFR (Figure 3b). After larval microinjection, fluorescence was checked under stereo-microscopy and then at high resolution by LSFM. Both TcI and TcII parasites were simultaneously detected attached to cardiac tissue in different structures. For instance, in one larva, two TcI trypomastigotes were attached to the atrioventricular valves, while a TcII trypomastigote adhered to the pericardium of the atrium (Video S4). Figure 4 provides a summary of the anatomic location of the trypomastigotes TcI and TcII detected in the heart structure of zebrafish larvae in all the experiments.

Finally, to establish the average number of parasites recovered after larvae injection, 20 fish larvae were injected: 18 with TcI parasites and 2 with 1× PBS as a control. After enzymatic digestion of the larvae, 5 out of 18 fish contained parasites, with an average of 2.6 parasites per larva. The highest parasite number found was nine parasites, whereas microinjection was calculated to have an input of 100 parasites per nL. The low number of detected parasites may be attributed to the large amount of injected solution, immediately ejected from the larvae after microinjection.

## 4. Discussion

The zebrafish model used here is versatile, cost-effective, space-saving, and relatively easy to handle. One of the advantages of this model is the easy visualization given the translucent properties of zebrafish, which makes it suitable for in vivo visualization of larvae through optical microscopy, using techniques such as LSFM. This advantage makes it an ideal in vivo model to study pathogen-host interactions, as shown in a previous study by our group, which provided visual evidence of the attachment of *T. cruzi* TcI trypomastigotes to heart tissue in zebrafish after microinjection [8]. The present study expands on this prior observation by documenting in vivo attachment of TcI and TcII parasites in zebrafish cardiac tissue, as well as the yolk sac and circulatory system. 

TcI and TcII are the main *T. cruzi* lineages, hence the importance of understanding their migration and adhesion patterns in vivo [5]. In our study, microinjected larvae survived long enough to observe the *T. cruzi* migration from the duct of Cuvier, which is the initial injection site, to the target tissue. Studies in humans have suggested a possible association between TcI infection, primarily in the cardiac tissue, and TcII infection in the gastrointestinal tract [6]. It was found that the TcI genotype circulates and then attaches to the cardiac tissue, which is consistent with the published literature in which chagasic heart disease is associated with this genotype [16]. Indeed, cytokine profile and heart disease are more associated with TcI chronic infection [17]. Here, TcII trypomastigotes attached to the cardiac structures of zebrafish larvae were found. As it is shown in the videos, the trypomastigote can be seen moving in synchrony with each heartbeat, indicating that the parasite adheres to the cardiac structures. To our knowledge, this is the first in vivo demonstration of TcII adhesion to heart tissue, and interestingly, TcII infection has been associated mainly with chronic mega-visceral Chagas disease [18]. Of note, TcII is easier to visualize than TcI (DA), because of the relatively larger size of the specific (isolate Y) parasite used. Remarkably, a high percentage of TcII parasites were found in the yolk sac, consistent with its association with digestive system infection. Gastrointestinal tissue attachment has previously been imaged in vivo in mice using bioluminescent *T. cruzi* [12].

The coexistence of both TcI and TcII in patients is poorly understood; however, in this in vivo model we document that both genotypes can be found attached to cardiac structures. This observation was confirmed by LSFM microscopy, where the parasite showed strong adherence to the heart, tolerating high blood flow driven by heartbeats. Given that, LSFM microscopy was executed more than 2 h after injection, parasites were attached for over 2 h, from their initial adhesion observed 30 to 60 min after injection. 

When a single injection was performed with both genotypes individually labeled and mixed in a single tube, only the TcI genotype parasites migrated along the zebrafish circulation. Multiple individual parasites were detected at the posterior ends of the larvae. Additionally, both the TcI and TcII genotypes were identified in cardiac tissue. In mixed parasitic infections, there is a likelihood of misidentification of minor parasite genotypes because populations that grow at a faster rate are more likely to be detected given the available typification approaches [19]. This could explain why the correlation between heart disease and infection with TcI is typically more prevalent in some regions, as it is often regarded as more infectious and pathogenic than TcII [6]. Interestingly, TcII was the most detected DTU in isolates from Brazilian patients with either cardiac, indeterminate, or cardio-digestive forms [20]. 

The reasons behind the parasite’s preference for a specific tissue are not fully understood but are likely to be multifactorial. Tissue attachment depends not only on the genetic variability of the parasite but also on host genetic factors, host immune system status [4], and parasite replication sites [21], which can even change during infection [1]. In a mouse model, *T. cruzi* showed identical tissue attachment in mice with the same H-2 haplotype, H-2^d^ (BALB/c and DBA-2 mice), but differed in H-2^b^ (C57BL/6 mice) using two different DTUs: Col 1.7G2 (TcI) and JG (TcII). In this case, it was suggested that major histocompatibility complex variability influenced the differential tissue distribution of each parasite variant [3].

Host factors, including lectins, particularly human galectin-3, a β-galactosyl-binding lectin, have been identified to play a role in the attachment and invasion of *T. cruzi* into dendritic and endothelial cells [22]. Cardiac attachment in *T. cruzi* seems to be influenced by the peptide motif FLY, which is conserved in all gp85/trans-sialidases and interacts with the vascular endothelium. Interestingly, this motif has greater avidity for cardiac vasculature than for vessels in other organs [10]. 

Cell surface glycosaminoglycans (GAGs), primarily heparan sulfate, play a crucial role in *T. cruzi* cell attachment and invasion [23]. In an in vitro model, heparan sulfate proteoglycans mediate the attachment of *T. cruzi* to cardiomyocytes [24]. Heparan sulfate is one of the most conserved molecules across species [25], and similar interactions with parasite proteins could occur during attachment to human and zebrafish hearts, although specific *T. cruzi* proteins involved have yet to be identified. Genomic comparisons between the human and zebrafish genomes have shown approximately 70% homology [26], and zebrafish have been used as a model for studying genetic cardiac diseases [27]. 

Although zebrafish are not commonly used as an infection model for certain pathogens, they are valuable for studying parasite attachment. In addition to *T. cruzi*, zebrafish models have been established for *Toxoplasma gondii* infection, demonstrating its interaction with macrophages and successful replication [28]. Lastly, it is essential to acknowledge that while the tissue niche of trypanosomiasis is relatively well known, a comprehensive understanding of the pathways governing its attachment is still lacking. Similarly, the invasion and pathogenesis of *Trypanosoma brucei* in the central nervous system remain largely unknown. A study with two-photon microscopy provided insights into specific tissue locations and the involvement of immune cells of this parasite during infection [29]. Moreover, despite advancements in understanding the cellular pathways facilitating parasite adherence to host cells, it is crucial to clarify that adherence does not necessarily imply invasion by the parasite [30].

These findings underscore the importance of further investigating the molecular mechanisms behind tissue specificity and attachment in *T. cruzi* infections. The zebrafish model, with its unique advantages, provides a promising platform for such studies, potentially leading to new insights into the pathogenesis and progression of Chagas disease. Future research could focus on identifying the specific host-parasite interactions that drive the differential tissue tropism observed in TcI and TcII, which may contribute to the development of targeted therapies aimed at preventing tissue-specific damage in infected individuals. 

## 5. Conclusions

Our study has provided evidence of the differential tissue migration and attachment of *T. cruzi* TcI and TcII genotypes within the zebrafish model, highlighting its utility for in vivo investigations of pathogen-host interactions. We document parasite adherence to multiple locations, not only within the heart but also the yolk sac and circulatory system, and the observation of cardiac involvement by both TcI and TcII DTUs after simultaneous injection. The simultaneous detection of TcI and TcII attachment to cardiac structures in zebrafish larvae offers new insights into the potential for co-infection. This model thus serves as a valuable tool for further dissecting the molecular mechanisms underlying tissue migration and attachment during *T. cruzi* infections and could aid in developing targeted therapeutic interventions.

## Figures and Tables

**Table 1:** Parasite injection outcome in zebrafish larvae by DTU TcI and TcII.

Parasite DTU	TcI	TcII	Both **
No. Injected larvae	81	64	33
**Injections**			
Unsuccessful	7 (9%)	12 (19%)	0 (0%)
Successful	74 (91%) *	52 (81%)	33 (70%)
Migration to organ	23 (28%)	18 (28%)	10 (30%)

* Values represent the total and percentage of injections where the parasite was detected. Success includes migration. ** Simultaneous injection of TcI and TcII trypomastigotes.

**Table 2:** Detection of parasite in organs according to DTU TcI and TcII.

Parasite DTU	TcI	TcII	Both **
No. Injected larvae *	74	52	33
**Injections**			
Eye/Brain	4 (17%)	3 (17%)	3 (30%)
Heart/Pericardium	8 (35%)	7 (39%)	4 (40%)
GI tract	5 (22%)	5 (28%)	1 (10%)
Circulation	6 (26%)	3 (17%)	2 (20%)

* Successful injections: parasite found inside the larvae. ** Simultaneous injection of TcI and TcII trypomastigotes.

## Data Availability

The data are presented in this paper.

## Supplementary Materials

The following supporting information can be downloaded at: https://www.mdpi.com/article/10.3390/pathogens14010025/s1, Video S1: Fluorescence Imaging of TcI Trypomastigote Migration in Zebrafish Larva, Video S2: TcI Trypomastigote Attachment to Zebrafish Cardiac Tissue Observed by Stereomicroscopy and LSFM, Video S3: TcII Trypomastigote Cardiac Attachment in Zebrafish Larva: Stereomicroscopy and LSFM Analysis, Video S4: Dual Imaging of TcI and TcII Trypomastigote Attachment in Zebrafish Cardiac Tissue using LSFM.

## References

[1] Pereira S.S., Trindade S., De Niz M., Figueiredo L.M. (2019). Tissue Tropism in Parasitic Diseases. Open Biol..

[2] de Souza W., de Carvalho T.M.U., Barrias E.S. (2010). Review on *Trypanosoma cruzi*: Host Cell Interaction. Int. J. Cell Biol..

[3] Andrade L.O., Machado C.R.S., Chiari E., Pena S.D.J., Macedo A.M. (2002). *Trypanosoma cruzi*: Role of Host Genetic Background in the Differential Tissue Distribution of Parasite Clonal Populations. Exp. Parasitol..

[4] Shelton W.J., Gonzalez J.M. (2024). Outcomes of patients in Chagas disease of the central nervous system: A systematic review. Parasitology.

[5] Tibayrenc M., Ayala F.J. (2022). Microevolution and subspecific taxonomy of *Trypanosoma cruzi*. Infect. Genet. Evol..

[6] Zingales B., Miles M.A., Campbell D.A., Tibayrenc M., Macedo A.M., Teixeira M.M.G., Schijman A.G., Llewellyn M.S., Lages-Silva E., Machado C.R. (2012). The Revised *Trypanosoma cruzi* Subspecific Nomenclature: Rationale, Epidemiological Relevance and Research Applications. Infect. Genet. Evol..

[7] Mateus J., Guerrero P., Lasso P., Cuervo C., González J.M., Puerta C.J., Cuéllar A. (2019). An Animal Model of Acute and Chronic Chagas Disease with the Reticulotropic Y Strain of *Trypanosoma cruzi* That Depicts the Multifunctionality and Dysfunctionality of T Cells. Front. Immunol..

[8] Akle V., Agudelo-Dueñas N., Molina-Rodriguez M.A., Kartchner L.B., Ruth A.M., González J.M., Forero-Shelton M. (2017). Establishment of Larval Zebrafish as an Animal Model to Investigate *Trypanosoma cruzi* Motility in vivo. J. Vis. Exp..

[9] Chatelain E., Konar N. (2015). Translational Challenges of Animal Models in Chagas Disease Drug Development: A Review. Drug Des. Dev. Ther..

[10] Tonelli R.R., Giordano R.J., Barbu E.M., Torrecilhas A.C., Kobayashi G.S., Langley R.R., Arap W., Pasqualini R., Colli W., Alves M.J.M. (2010). Role of the Gp85/Trans-Sialidases in *Trypanosoma cruzi* Tissue Tropism: Preferential Binding of a Conserved Peptide Motif to the Vasculature in Vivo. PLoS Negl. Trop. Dis..

[11] Pérez-Mazliah D., Ward A.I., Lewis M.D. (2021). Host-Parasite Dynamics in Chagas Disease from Systemic to Hyper-Local Scales. Parasite Immunol..

[12] Silberstein E., Serna C., Fragoso S.P., Nagarkatti R., Debrabant A. (2018). A Novel Nanoluciferase-Based System to Monitor *Trypanosoma cruzi* Infection in Mice by Bioluminescence Imaging. PLoS ONE.

[13] Dóró É., Jacobs S.H., Hammond F.R., Schipper H., Pieters R.P., Carrington M., Wiegertjes G.F., Forlenza M. (2019). Visualizing Trypanosomes in a Vertebrate Reveals Novel Swimming Behaviours, Adaptations and Attachment Mechanisms. eLife.

[14] Schneider C.A., Rasband W.S., Eliceiri K.W. (2012). NIH Image to ImageJ: 25 Years of Image Analysis. Nat. Methods.

[15] Vargas-Patron L.A., Agudelo-Dueñãs N., Madrid-Wolff J., Venegas J.A., González J.M., Forero-Shelton M., Akle V. (2019). Xenotransplantation of Human Glioblastoma in Zebrafish Larvae: In Vivo Imaging and Proliferation Assessment. Biol. Open.

[16] Messenger L.A., Miles M.A., Bern C. (2015). Between a Bug and a Hard Place: *Trypanosoma cruzi* Genetic Diversity and the Clinical Outcomes of Chagas Disease. Expert. Rev. Anti Infect. Ther..

[17] Poveda C., Fresno M., Gironès N., Martins-Filho O.A., Ramírez J.D., Santi-Rocca J., Marin-Neto J.A., Morillo C.A., Rosas F., Guhl F. (2014). Cytokine profiling in Chagas disease: Towards understanding the association with infecting *Trypanosoma cruzi* discrete typing units (a BENEFIT TRIAL sub-study). PLoS ONE.

[18] Cura C.I., Lucero R.H., Bisio M., Oshiro E., Formichelli L.B., Burgos J.M., Lejona S., Brusés B.L., Hernández D.O., Severini G.V. (2012). *Trypanosoma cruzi* Discrete Typing Units in Chagas Disease Patients from Endemic and Non-Endemic Regions of Argentina. Parasitology.

[19] Ledezma A.P., Blandon R., Schijman A.G., Benatar A., Saldaña A., Osuna A. (2020). Mixed Infections by Different *Trypanosoma cruzi* Discrete Typing Units among Chagas Disease Patients in an Endemic Community in Panama. PLoS ONE.

[20] Nielebock M.A.P., Moreira O.C., das Chagas Xavier S.C., de Freitas Campos Miranda L., de Lima A.C.B., de Jesus Sales Pereira T.O., Hasslocher-Moreno A.M., Britto C., Sangenis L.H.C., Saraiva R.M. (2020). Association between *Trypanosoma cruzi* DTU TcII and Chronic Chagas Disease Clinical Presentation and Outcome in an Urban Cohort in Brazil. PLoS ONE.

[21] McCall L.I., Siqueira-Neto J.L., McKerrow J.H. (2016). Location, Location, Location: Five Facts about Tissue Tropism and Pathogenesis. PLoS Pathog..

[22] Vray B., Camby I., Vercruysse V., Mijatovic T., Bovin N.V., Ricciardi-Castagnoli P., Kaltner H., Salmon I., Gabius H.J., Kiss R. (2004). Up-Regulation of Galectin-3 and Its Ligands by *Trypanosoma cruzi* Infection with Modulation of Adhesion and Migration of Murine Dendritic Cells. Glycobiology.

[23] de Oliveira F.O.R., Alves C.R., Calvet C.M., Toma L., Bouças R.I., Nader H.B., de Castro Côrtes L.M., Krieger M.A., Meirelles M.d.N.S.L., de Souza Pereira M.C. (2008). *Trypanosoma cruzi* Heparin-Binding Proteins and the Nature of the Host Cell Heparan Sulfate-Binding Domain. Microb. Pathog..

[24] Calvet C.M., Toma L., De Souza F.R., De Meirelles M.D.N.S.L., Pereira M.C.S. (2003). Heparan Sulfate Proteoglycans Mediate the Invasion of Cardiomyocytes by *Trypanosoma cruzi*. J. Eukaryot. Microbiol..

[25] Esko J.D., Lindahl U. (2001). Molecular Diversity of Heparan Sulfate. J. Clin. Investig..

[26] Howe K., Clark M.D., Torroja C.F., Torrance J., Berthelot C., Muffato M., Collins J.E., Humphray S., McLaren K., Matthews L. (2013). The Zebrafish Reference Genome Sequence and Its Relationship to the Human Genome. Nature.

[27] Tu S., Chi N.C. (2012). Zebrafish Models in Cardiac Development and Congenital Heart Birth Defects. Differentiation.

[28] Yoshida N., Domart M.C., Peddie C.J., Yakimovich A., Mazon-Moya M.J., Hawkins T.A., Collinson L., Mercer J., Frickel E.M., Mostowy S. (2020). The Zebrafish as a Novel Model for the in Vivo Study of *Toxoplasma gondii* Replication and Interaction with Macrophages. DMM Dis. Models Mech..

[29] Coles J.A., Myburgh E., Ritchie R., Hamilton A., Rodgers J., Mottram J.C., Barrett M.P., Brewer J.M. (2015). Intravital Imaging of a Massive Lymphocyte Response in the Cortical Dura of Mice after Peripheral Infection by Trypanosomes. PLoS Negl. Trop. Dis..

[30] Salassa B.N., Romano P.S. (2019). Autophagy: A Necessary Process during the *Trypanosoma cruzi* Life-Cycle. Virulence.

