# Supplementary material for: In Vivo Imaging of Cardiac Attachment of TcI and TcII Variants of Trypanosoma cruzi in a Zebrafish Model
Source: Pathogens. 2025 Jan 1;14(1):25. doi: 10.3390/pathogens14010025 (PMC11769151; doi:10.3390/pathogens14010025)
Supplement: Supplementary file 1 [file pathogens-14-00025-s001.zip › Supporting information (videos).pdf]

Supporting information

Supplementary video S1**.** Zebrafish larva under stereomicroscope 4x after injection into the duct of Cuvier. Migration of a TcI trypomastigote is observed by fluorescence from the site of injection to the cardiac circulation towards the cardinal vein. Then, larval anatomy was observed in the bright field. Yellow arrow indicates parasitic mobility in the larval blood vessel.

https://figshare.com/s/af3b2c6294d6c42b19bb

Supplementary video S2. Zebrafish larvae injected with TcI trypomastigotes were observed under fluorescence stereomicroscopy. The yellow arrow shows the trypomastigote attached to the cardiac tissue moving with each heartbeat. The anatomy of the larva was then observed in the bright field. Finally, the same larva was observed 4 h after microinjection under LSFM showing one CTFR-labelled TcI trypomastigote attached to the atrioventricular valve.

https://figshare.com/s/36bbee500abb4be0ba4c

Supplementary video S3**.** Zebrafish larvae injected with TcII trypomastigotes were observed under fluorescence stereomicroscopy. The yellow arrow shows the trypomastigote attached to the cardiac tissue moving with each heartbeat. Then, under transmitted light, larval anatomy is observed. Finally, the same larva was observed 4 h after microinjection under LSFM showing three CTFR-labelled TcII trypomastigotes with two attached to the atrioventricular valve, and one attached to the pericardium of the atrium.

https://figshare.com/s/e84d97e47409f5b5dd09

***Supplementary video S4*.** Zebrafish larva injected with both TcI and TcII DTUs, ventral view under LSFM. On the right in red, CTRF-labelled TcI trypomastigotes can be seen attached to atrioventricular valves. On the left in green, one CTCFSE-labelled TcII trypomastigotes adhered to the pericardium on the atrium.

https://figshare.com/s/5889494175356d3e5f0d
